# Significance of lactate clearance in septic shock patients with high bilirubin levels

**DOI:** 10.1038/s41598-021-85700-w

**Published:** 2021-03-18

**Authors:** Nozomi Takahashi, Taka-aki Nakada, Keith R. Walley, James A. Russell

**Affiliations:** 1 Department of Emergency and Critical Care Medicine, Chiba University Graduate School of Medicine, 1-8-1 Inohana, Chuo, Chiba 260-8677 Japan; 2 Centre for Heart Lung Innovation, University of British Columbia, Vancouver, Canada

**Keywords:** Prognostic markers, Bacterial infection, Liver diseases

## Abstract

Lactate clearance is affected by hepatic function. However, it is unclear whether the association between hepatic dysfunction and lactate clearance can act as a prognostic marker of clinical outcomes in patients with septic shock. We aimed to evaluate the association between lactate clearance and mortality in two cohorts of septic shock patient who had hepatic dysfunction based on their total serum bilirubin levels (TBIL). Lactate clearance at 24 h after the onset of septic shock was analyzed using two cohorts, sub-categorized into two groups based on TBIL: < 2 mg/dL and ≥ 2 mg/dL. In the derivation cohort, lactate clearance was lower in non-survivors than in survivors with TBIL ≥ 2 mg/dL, while there was no significant difference in lactate clearance between non-survivors and survivors with TBIL < 2 mg/dL. Multivariate logistic regression analysis revealed that increased lactate clearance was significantly associated with decreased 28-day mortality in the TBIL ≥ 2 mg/dL group (10% lactate clearance, adjusted odds ratio [OR]: 0.88, 95% confidence interval (CI): 0.80–0.97, *P* = 0.0075), Creatinine level ≥ 2 mg/dL group (adjusted OR: 0.88, 95% CI: 0.81–0.95, *P* = 0.00069) and APACHE II score ≥ 35 group (adjusted OR: 0.93, 95% CI: 0.87–0.98, *P* = 0.013). In the validation cohort, lactate clearance was lower in non-survivors than in survivors with TBIL ≥ 2 mg/dL, while no significant difference in lactate clearance was observed between non-survivors and survivors with TBIL < 2 mg/dL. Increased lactate clearance was significantly associated with decreased 28-day mortality in the TBIL ≥ 2 mg/dL group (10% lactate clearance, adjusted OR: 0.89, 95% CI: 0.83–0.96, *P* = 0.0038) and the association was just about significant in APACHE II score ≥ 35 group (adjusted OR: 0.86, 95% CI: 0.74–1.00, *P* = 0.051). In conclusion, increased lactate clearance in septic shock patients with hepatic dysfunction (TBIL ≥ 2 mg/dL) or high severity (APACHE II score ≥ 35) was associated with decreased 28-day mortality.

## Introduction

Blood lactate levels potentially reflect the imbalance between oxygen delivery and consumption during global tissue hypoxia, which reduces the availability of pyruvate for the tricarboxylic acid (TCA) cycle and accelerates aerobic glycolysis as a result of excess beta-adrenergic stimulation^1,2^. Blood lactate level is the cornerstone of diagnosis and management of patients with septic shock^3–6^. Septic shock patients with increased blood lactate levels have poor clinical outcomes; therefore, persistently elevated lactate due to decreased lactate clearance may be a danger signal for the management of septic shock^7–10^.

Blood lactate level indicates the balance between lactate production and lactate clearance^11,12^. Hepatic function is key to eliminating lactate, while hepatic dysfunction potentially decreases lactate clearance^13,14^. Lactate clearance has been extensively investigated in septic shock^7–9,15,16^; however, it remains unknown whether the association between lactate clearance and clinical outcomes in patients with septic shock differs between patients with and without hepatic dysfunction.

Thus, we hypothesized that hepatic function would influence the relationship between lactate clearance and mortality in septic shock. We investigated the association between lactate clearance and 28-day mortality in two cohorts of septic shock patients who were sub-divided into two groups based on blood total bilirubin levels (TBIL) indicating hepatic dysfunction. We also determined changes in the anion gap and base excess, which directly reflect the amount of nonvolatile acid produced during tissue hypoxia and may be affected by hepatic function.

## Materials and methods

### Study design, definition, and patients

This was a retrospective observational study. Septic shock was characterized by the presence of systemic inflammatory response syndrome caused by infection^17^, at least one new organ dysfunction according to the Brussels criteria, and persistent hypotension despite adequate fluid resuscitation^18^. This study utilized the previous definition of septic shock (Sepsis-2) because the current one (Sepsis-3) includes hepatic dysfunction assessed by the Sequential Organ Failure Assessment (SOFA) score, and one of the cohorts we used for validation was assessed using the Sepsis-2 definition. According to these criteria, hepatic dysfunction was defined as TBIL ≥ 2 mg/dL^19^. We selected TBIL for the assessment of hepatic dysfunction as opposed to albumin levels and coagulation tests because they can be affected by nutrition and coagulation disorders in patients with septic shock. The need to obtain informed consent from the patients was explicitly waived by the institutional review board which approved this study’s protocols.

### Derivation cohort: CHIBA cohort

We screened total 9290 patients who were admitted to the intensive care unit (ICU) at Chiba University Hospital (CHIBA), Chiba, Japan between October 2012 and September 2018. The ICU had 22 beds and was utilized by patients following emergency room admission and after elective surgery, accounting for approximately 80% of ICU beds. Of 9290 patients, 230 were diagnosed with septic shock on ICU admission and had blood lactate clearance, delta anion gap, and delta base excess data available; these patients were subsequently included in the analysis. This study was approved by the Institutional Review Board of Chiba University Graduate School of Medicine and was performed in accordance with the committee’s guidelines; the requirement to obtain informed consent was waived.

### Validation cohort: VASST cohort

The Vasopressin and Septic Shock Trial (VASST) was a multicenter (27 ICUs in Canada, Australia, and the United States), randomized, double-blind trial conducted between July 2001 and April 2006^18^. Of 6229 patients, 396 were screened for septic shock in the VASST and were included in our analyses. The Institutional Review Board of St. Paul’s Hospital and the University of British Columbia (UBC) approved the study, which was performed in accordance with the committee’s guidelines. The requirement for informed consent was waived in accordance with the ethical standards of the local institutional review board.

### Measurements and lactate clearance

Blood lactate level, base excess, and anion gap were measured on the day patients presented with septic shock (day 1) as well as 24 h later (day 2). Blood lactate clearance (%) was calculated using the following formula^7–9^:

$${\text{Lactate}}\,{\text{clearance}}\,\left( \% \right)\, = \,\left( {{\text{Lactate}}^{{{\text{day}}1}} {-}{\text{Lactate}}^{{{\text{day}}2}} } \right)/{\text{Lactate}}^{{{\text{day}}1}} *100$$

where Lactate^day1^ = lactate level at the diagnosis of septic shock and Lactate^day2^ = lactate level 24 h after the diagnosis of septic shock.

The anion gap was calculated using the concentrations of sodium, chloride, and bicarbonate ions and was available only in the derivation cohort. Delta base excess and anion gap were calculated by subtracting day 2 values from day 1 values.

### Statistical analyses

The primary outcome variable was lactate clearance. The secondary outcome variables were delta base excess and delta anion gap. Patients were categorized into two groups based on their TBIL on day 1: < 2 mg/dL and ≥ 2 mg/dL.

We used logistic regression analysis to test the association between lactate clearance and 28-day mortality in patients with TBIL < 2 mg/dL or ≥ 2 mg/dL. Multivariate analysis was performed to adjust for potential baseline imbalances (age, sex, and Acute Physiology and Chronic Health Evaluation II (APACHE II) score), as reported previously^9,20–22^. Creatinine (Cre) level (< and ≥ 2 mg/dL), platelet count (< and ≥ 80 × 10^3^/mm^3^), and APACHE II score (< and ≥ 35) were also analyzed to evaluate the association between severity and organ dysfunction. Cutoff values were derived according to the Brussels criteria and using the Youden’s index. The correlation between lactate clearance and delta anion gap was analyzed using the Pearson correlation coefficient. Different TBIL cutoff values of 1 mg/dL, 3 mg/dL, and 4 mg/dL were tested using multivariate analysis to determine the appropriate threshold. The cutoff value of lactate clearance was determined to be 10% using the derivation cohort as per the Youden’s index.

Univariate analysis was performed using the Mann–Whitney U test. A two-tailed *P* value of < 0.05 was considered statistically significant. Data are presented as medians with interquartile range. All analyses were performed using R version 3.3.3 (R Foundation for Statistical Computing, Vienna, Austria, http://www.R-roject.org/) and PRISM version 8 (GraphPad Software Inc, California, USA).

### Ethics approval and consent to participate

This study was approved by the Institutional Review Board of Chiba University Graduate School of Medicine, the Institutional Review Board of St. Paul’s Hospital and the University of British Columbia. The requirement to obtain written informed consent was waived because of the study design.

## Results

Of 230 patients in the derivation cohort, 153 had TBIL < 2 mg/dL, and 77 had TBIL ≥ 2 mg/dL (Table 1). Baseline characteristics indicated no significant difference between patients with TBIL ≥ 2 mg/dL and TBIL < 2 mg/dL although lactate level on day 1 was higher in the TBIL ≥ 2 mg/dL group than that in the TBIL ≥ 2 mg/dL group.

**Table 1:** Baseline characteristics, vital signs, blood gas analysis and laboratory data in the derivation cohort (CHIBA cohort, n = 230).

	TBIL < 2 mg/dL (n = 153)	TBIL** ≥ **2 mg/dL (n = 77)	*P* value
Age, year	69 (59, 76)	66 (56, 74)	0.19
Male sex, n (%)	91 (59.5)	50 (64.9)	0.51
APACHE II score^a^	31 (26, 36)	34 (24, 40)	0.20
**Comorbidity, n (%)**			
Congestive heart failure	15 (9.8)	12 (15.6)	0.29
Chronic pulmonary disease	7 (4.6)	2 (2.6)	0.71
End stage renal failure	11 (7.2)	9 (11.7)	0.37
Hepatic cirrhosis	7 (4.6)	9 (11.7)	0.084
Steroid use (> 7 days)	25 (16.3)	8 (10.4)	0.31
**Vital signs on Day1**			
Body temperature, °C	37.0 (36.5, 38.2)	37.4 (36.6, 38.3)	0.68
Heart rate, beats/min	108 (94, 124)	114 (98, 129)	0.11
Mean arterial pressure, mmHg	63 (55, 77)	66 (56, 80)	0.50
**Blood gas analysis on Day1**			
pH	7.36 (7.28, 7.44)	7.36 (7.29, 7.45)	0.50
PaCO_2_, mmHg	35 (29, 44)	35 (29, 41)	0.41
PaO_2_, mmHg	99 (73, 147)	97 (74, 154)	0.85
Base excess, mmol/L	− 4.6 (− 8.2, 0.3)	− 3.8 (− 9.0, − 0.2)	0.99
Anion gap, mEq/L	14.5 (11.8, 19.0)	18.0 (12.0, 23.5)	0.21
Lactate, mmol/L	2.8 (1.5, 4.9)	3.6 (1.9, 7.0)	0.031
**Laboratory data on Day1**			
WBC, *10^3^/mm^3^	10.8 (34, 171)	11.1 (62, 166)	0.52
Platelet, *10^5^/mm^3^	13.2 (6.9, 23.1)	7.7 (3.6, 12.7)	< 0.0001
Total bilirubin, mg/dL	0.9 (0.6, 1.1)	3.6 (2.7, 6.1)	< 0.0001
Creatinine, mg/dL	1.67 (1.04, 2.57)	1.81 (1.14, 3.03)	0.28
Lactate level on Day2, mmol/L	1.7 (1.2, 2.6)	2.2 (1.5, 3.6)	0.0023
Delta lactate Day1-2, mmol/L^b^	0.8 (0.0, 2.7)	0.7 (0.0, 2.8)	0.74
Lactate clearance Day1–2^c^	34.7 (0.0, 61.9)	35.3 (0.0, 53.9)	0.51
Delta base excess Day1–2^d^	− 3.8 (− 8.2, -0.7)	− 3.7 (− 9.5, -0.9)	0.97
Delta anion gap Day1–2^e^	− 4 (− 8, − 3)	− 4 (− 8, 3)	0.43
28 Days survival, n (%)	125 (81.7)	60 (77.9)	0.61

Median (inter quartile range).

*P* values were calculated using Pearson’s chi-square test and Mann–Whitney U test.

^a^APACHE, acute physiology and chronic health evaluation.

^b^Delta lactate was calculated by subtracting lactate level on day2 from day1.

^c^Lactate clearance was calculated by delta lactate divided by day1 value.

^d^Delta base excess was calculated by subtracting base excess on day1 from day2.

^e^Delta anion gap was calculated by subtracting anion gap on day1 from day2.

**Figure 1:**
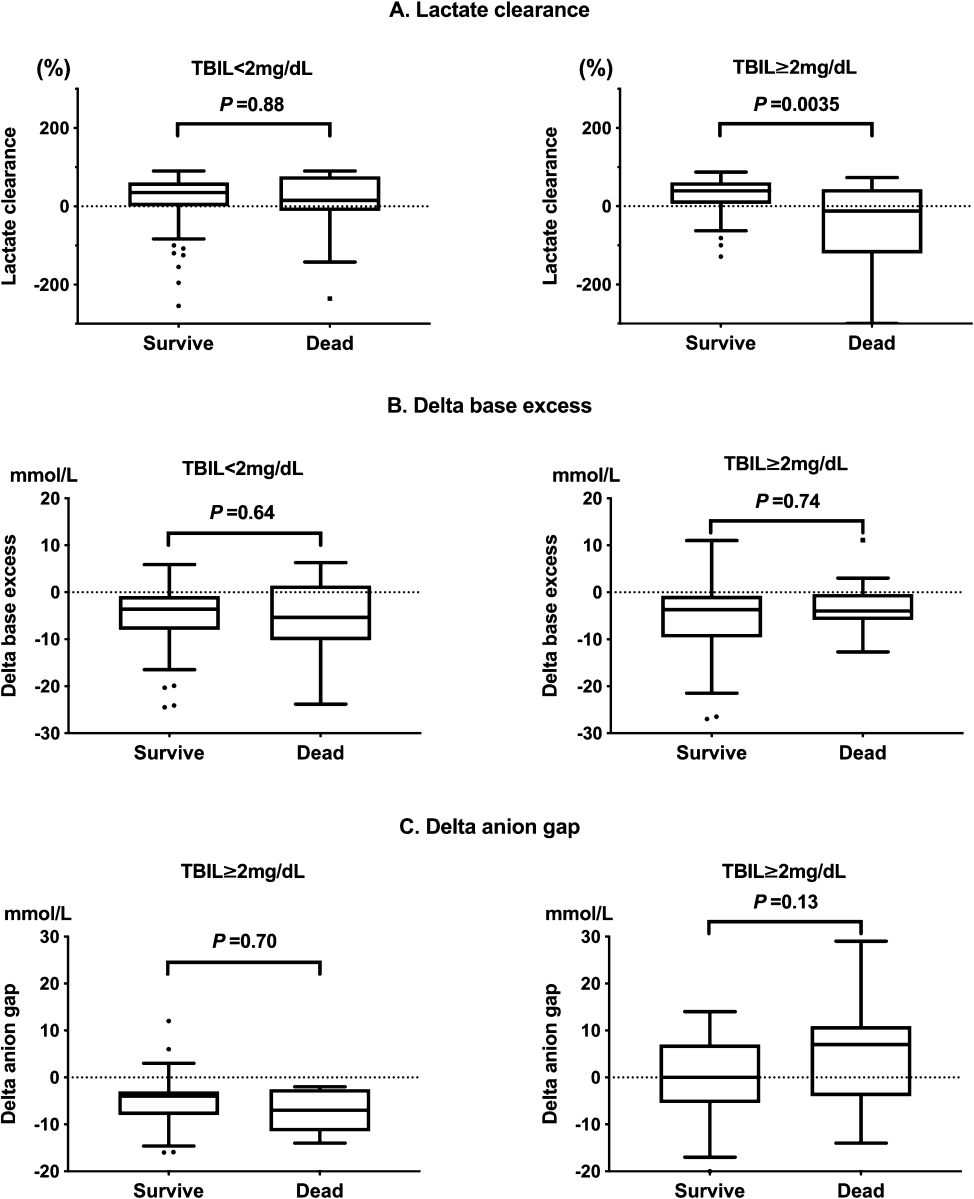
Comparisons of (**A**) lactate clearance, (**B**) delta base excess, and (**C**) delta anion gap between survivors and non-survivors in the derivation cohort.

In the TBIL ≥ 2 mg/dL group, non-survivors had significantly lower lactate clearance compared with survivors (*P* = 0.0035). However, there was no significant difference in lactate clearance between survivors and non-survivors in the TBIL < 2 mg/dL group (*P* = 0.88) (Fig. 1A). Lactate clearance was not significantly correlated with TBIL (TBIL ≥ 2 mg/dL group, *P* = 0.82; TBIL < 2 mg/dL group, *P* = 0.35; total, *P* = 0.80). There were no significant differences in delta base excess between non-survivors and survivors in both the TBIL ≥ 2 mg/dL (*P* = 0.74) and TBIL < 2 mg/dL (*P* = 0.64) groups. No significant differences were found in delta anion gap between non-survivors and survivors in the TBIL < 2 mg/dL group (*P* = 0.70). However, a non-significant trend of lower delta anion gap was observed in survivors compared to that in non-survivors in the TBIL ≥ 2 mg/dL group (*P* = 0.13) (Fig. 1B,C). Lactate clearance was significantly correlated with delta anion gap in the derivation cohort (all, R = 0.54, *P* < 0.0001; TBIL < 2 mg/dL, R = 0.56, *P* = 0.00017; TBIL ≥ 2 mg/dL, R = 0.57, *P* < 0.0001) (eFigure 1, Supplemental Digital Content).

Multivariate logistic regression analysis with adjustments for baseline imbalances revealed that patients with increased lactate clearance had significantly decreased 28-day mortality in the TBIL ≥ 2 mg/dL group (10% lactate clearance, adjusted odds ratio [OR]: 0.88, 95% confidence interval [CI]: 0.80–0.97, *P* = 0.0075, absolute risk difference: 25%, 95% CI: 4–46%). However, lactate clearance was not significantly associated with altered 28-day mortality in the TBIL < 2 mg/dL group in the derivation cohort (10% lactate clearance, adjusted OR: 0.99, 95% CI: 0.93–1.05, *P* = 0.69) (Table 3A). Similar to lactate clearance, patients with an increased delta anion gap had significantly decreased 28-day mortality in the TBIL ≥ 2 mg/dL group (delta anion gap, adjusted OR: 1.09, 95% CI: 1.00–1.19, *P* = 0.045). However, delta anion gap was not significantly associated with altered 28-day mortality in the TBIL < 2 mg/dL group (delta anion gap, adjusted OR: 0.96, 95% CI: 0.83–1.11, *P* = 0.58) (Table 3B). Delta base excess was also not significantly associated with altered 28-day mortality in the multivariate logistic regression analysis following the same adjustments for baseline imbalances (delta base excess, TBIL < 2 mg/dL, *P* = 0.65; TBIL ≥ 2 mg/dL, *P* = 0.74). Analysis of different TBIL cutoff values revealed that decreased lactate clearance was most relevant to 28-day mortality at a cutoff value of 2 mg/dL (eTable 1, Supplemental Digital Content).

In the Cre ≥ 2 mg/dL group, non-survivors had significantly lower lactate clearance compared to survivors (*P* = 0.0006). However, there was no significant difference in lactate clearance between survivors and non-survivors in the Cre < 2 mg/dL group (*P* = 0.71) (eFigure 2A, Supplemental Digital Content). Non-survivors had significantly lower lactate clearance compared to survivors (*P* = 0.03), in the APACHE II score ≥ 35 group, while there was no significant difference in lactate clearance between survivors and non-survivors in the APACHE II < 35 group (*P* = 0.84) (eFigure 2B, Supplemental Digital Content).

Patients with increased lactate clearance had a significantly decreased 28-day mortality in the Cre ≥ 2 mg/dL group (10% lactate clearance, adjusted OR: 0.88, 95% CI: 0.81–0.95, *P* = 0.00069) and APACHE II score ≥ 35 group (10% lactate clearance, adjusted OR: 0.93, 95% CI: 0.87–0.98, *P* = 0.013). However, lactate clearance was not significantly associated with altered 28-day mortality in both the Cre < 2 mg/dL group (10% lactate clearance, adjusted OR: 1.01, 95% CI: 0.94–1.08, *P* = 0.76) and APACHE II score < 35 group (10% lactate clearance, adjusted OR: 0.96, 95% CI: 0.89–1.04, *P* = 0.37) in the derivation cohort (eTable 2, Supplemental Digital Content). Both platelet count and the APACHE II score were not significantly associated with altered 28-day mortality in the derivation cohort.

**Figure 2:**
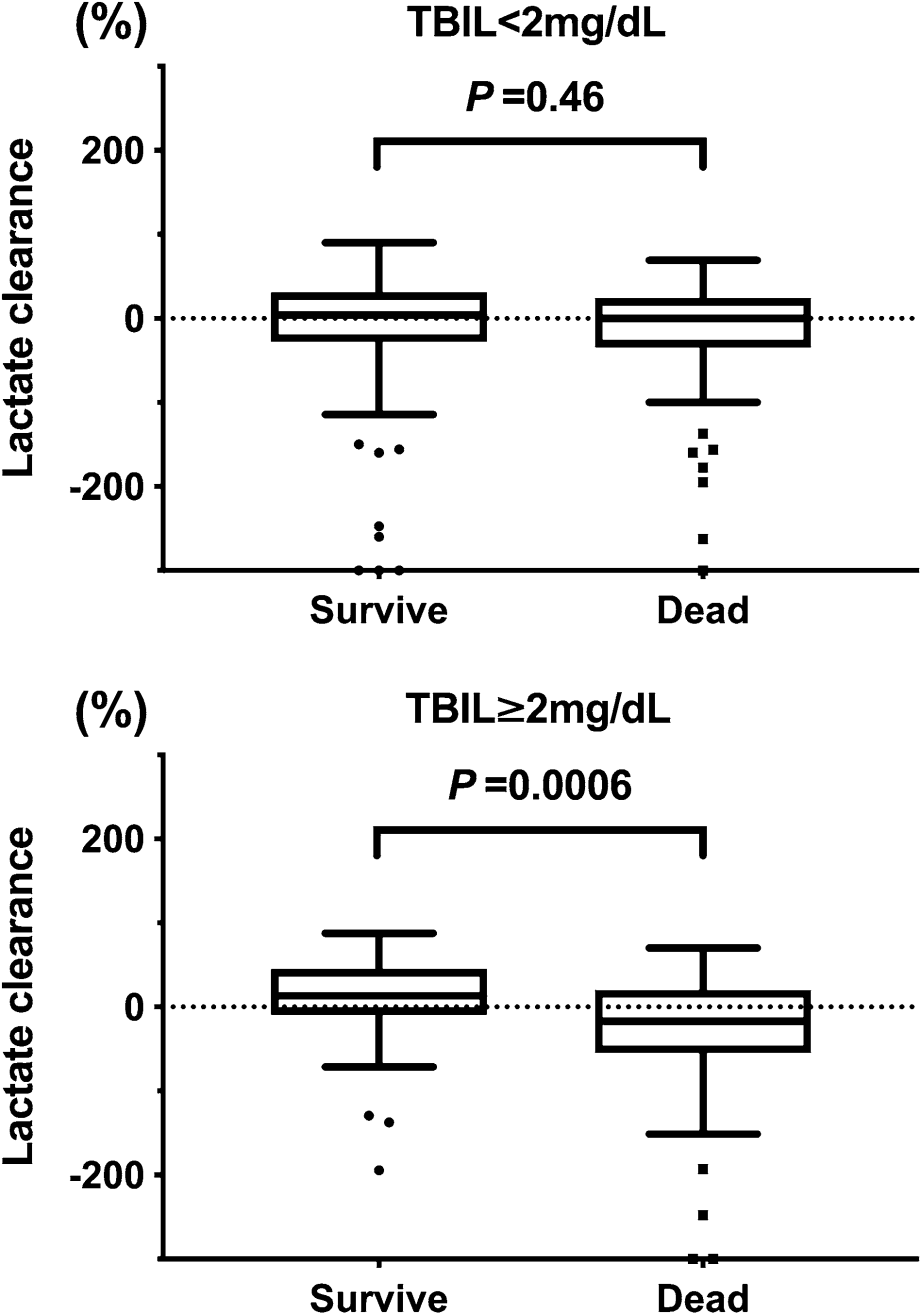
Comparison of lactate clearance between survivors and non-survivors in the validation cohort.

We then analyzed lactate clearance in the TBIL ≥ 2 mg/dL group using the validation cohort (Table 2). In terms of baseline characteristics, patients in the TBIL ≥ 2 mg/dL group were younger and had an increased probability of chronic hepatic failure compared to those in the TBIL < 2 mg/dL group. Similar to the derivation cohort, non-survivors had significantly lower lactate clearance compared to survivors in the TBIL ≥ 2 mg/dL group (*P* = 0.0006), while no significant difference in lactate clearance was observed between non-survivors and survivors in the TBIL < 2 mg/dL group (*P* = 0.46) (Fig. 2). Furthermore, patients with increased lactate clearance had significantly decreased 28-day mortality in the TBIL ≥ 2 mg/dL group (10% lactate clearance, adjusted OR: 0.89, 95% CI: 0.83–0.96, *P* = 0.0038, absolute risk difference: 20%, 95% CI: 2–37%). However, in the TBIL < 2 mg/dL group, lactate clearance was not significantly associated with altered 28-day mortality in the validation cohort (Table 3C).

**Table 2:** Baseline characteristics, vital signs, blood gas analysis and laboratory data in the derivation cohort (VASST cohort, n = 396).

	TBIL < 2 mg/dL (n = 280)	TBIL** ≥ **2 mg/dL (n = 116)	*P* value
Age, year	65 (51, 74)	57 (45, 66)	< 0.00001
Male sex, n (%)	161 (57.5)	63 (54.3)	0.64
APACHE II score	26 (22, 32)	28 (23, 33)	0.094
**Comorbidity, n (%)**			
Chronic heart failure	18 (6.4)	10 (8.6)	0.58
Chronic pulmonary disease	52 (18.5)	8 (6.9)	0.0052
Chronic hepatic failure	16 (5.7)	36 (31.0)	< 0.00001
End stage renal failure	34 (12.1)	13 (11.2)	0.93
Chronic steroid use	62 (22.1)	29 (25.0)	0.63
**Vital signs on Day1**			
Body temperature, °C^a^	38.6 (37.7, 39.3)	38.4 (37.6, 39.2)	0.37
Heart rate, beats/min^b^	125 (108, 140)	130 (118, 140)	0.20
Mean arterial pressure, mmHg^c^	54 (50, 60)	55 (49, 61)	0.61
**Laboratory data on Day1**			
WBC, *10^3^/mm^3^	14.2 (9.2, 21.5)	13.6 (7.5, 18.7)	0.076
Platelet, *10^5^/mm^3^	164 (92, 262)	92 (45, 149)	< 0.00001
Total bilirubin, mg/dL	0.8 (0.5, 1.3)	3.8 (2.6, 6.8)	< 0.00001
Creatinine, mg/dL	1.70 (1.06, 2.91)	2.18 (1.36, 3.31)	0.030
Lactate level on Day1, mmol/L	2.2 (1.3, 3.8)	3.5 (1.9, 6.5)	< 0.00001
Lactate level on Day2, mmol/L	1.9 (1.3, 3.0)	3.2 (1.8, 5.6)	< 0.00001
Delta lactate Day1-2, mmol/L	0.1 (− 0.5, 0.7)	0.2 (− 0.8, 1.4)	0.56
Lactate clearance Day1–2	0.8 (− 28.6, 29.7)	6.2 (− 27.1, 33.0)	0.62
28 Days survival, n (%)	184 (65.7)	69 (59.4)	0.29

Median (inter quartile range).

*P* values were calculated using Pearson’s chi-square test and Mann–Whitney U test.

^a^Most abnormal degree on Day1.

^b^Highest rate on Day1.

^c^Lowest pressure on Day1.

**Table 3:** Multivariate logistic regression analysis for 28 days mortality.

	TBIL < 2 mg/dL		TBIL** ≥ **2 mg/dL	
	Odd ratio (95%CI)	*P* value	Odd ratio (95%CI)	*P* value
**A. Derivation cohort, lactate clearance**				
Age-per year	0.98 (0.95–1.01)	0.17	1.03 (0.95–1.11)	0.45
Male sex	0.78 (0.31–1.91)	0.58	1.26 (0.30–5.22)	0.76
APACHE II score	1.13 (1.06–1.22)	0.00043	1.03 (0.95–1.11)	0.45
Lactate clearance -per 10%	0.99 (0.93–1.05)	0.69	0.88 (0.80–0.97)	0.0075
**B. Derivation cohort, delta anion gap**				
Age -per year	0.97 (0.90–1.04)	0.38	1.05 (0.99–1.12)	0.13
Male sex	0.32 (0.037–2.75)	0.30	2.15 (0.41–11.2)	0.37
APACHE II score	1.11 (0.96–1.28)	0.15	1.11 (1.00–1.24)	0.05
Delta anion gap	0.96 (0.83–1.11)	0.58	1.09 (1.00–1.19)	0.045
**C. Validation cohort**				
Age-per year	1.01 (0.99–1.02)	0.57	1.02 (0.99–1.05)	0.12
Male sex	0.97 (0.57–1.65)	0.91	1.03 (0.44–2.38)	0.95
APACHE II score	1.12 (1.08–1.17)	< 0.0001	1.09 (1.03–1.16)	0.0043
Lactate clearance-per 10%	1.01 (0.99–1.03)	0.48	0.89 (0.83–0.96)	0.0038

APACHE, acute physiology and chronic health evaluation.

Non-survivors had significantly lower lactate clearance compared to survivors in the Cre ≥ 2 mg/dL group (*P* = 0.049), while no significant difference in lactate clearance was observed in the Cre < 2 mg/dL group (*P* = 0.12) (eFigure 3A, Supplemental Digital Content). In the APACHE II score ≥ 35 group, non-survivors had significantly lower lactate clearance compared to survivors (*P* = 0.02). However, there was no significant difference in lactate clearance between survivors and non-survivors in the Cre < 2 mg/dL group (*P* = 0.071) (eFigure 3B, Supplemental Digital Content).

In both the Cre ≥ 2 mg/dL and APACHE II score ≥ 35 groups, lactate clearance was not significantly associated with altered 28-day mortality in the validation cohort (eTable 3, Supplemental Digital Content).

## Discussion

In this study, we investigated lactate clearance in septic shock patients, stratified according to hepatic dysfunction. The results of this study showed that increased lactate clearance during the initial 24 h was significantly associated with decreased mortality in septic shock patients with TBIL ≥ 2 mg/dL. In contrast, lactate clearance was not associated with altered mortality in patients with TBIL < 2 mg/dL.

Lactate clearance has previously been investigated as a potential prognostic marker for sepsis/septic shock^7,9,15,20^. However, increased lactate clearance is not always associated with decreased mortality in sepsis/septic shock. In this study including two septic shock cohorts, the association between increased lactate clearance and decreased 28-day mortality was significant in the TBIL ≥ 2 mg/dL group but not in the TBIL group < 2 mg/dL; this may explain the inconsistent conclusions of previous studies in mixed patient populations concerning hepatic dysfunction. The present study highlights the importance of hepatic dysfunction when considering lactate clearance as a prognostic marker.

Several studies have investigated lactate kinetics^23–27^, which is a complicated topic, and blood lactate level depends on the rate of production and removal of lactate from the blood as well as cell metabolism. Since sustained hyperlactatemia or even a rise in lactate levels could potentially reflect decreased lactate clearance rather than an increase in lactate production in septic shock patients, hepatic function could be important for the elimination of lactate^28^. A study using intravenous infusion of sodium lactate revealed that the clearance of lactate from the blood in normal subjects in the postprandial state was 17.9 mL/kg/min^23^; it decreased to 14.5 mL/kg/min in patients with hepatic dysfunction^24^. Additionally, due to impaired hepatic lactate uptake, lactate half-life was significantly longer in patients with than that in patients without liver cirrhosis (18.8 min vs. 14.7 min)^25^. Similarly, in severe sepsis/septic shock, hepatic dysfunction was significantly associated with impaired lactate clearance and normalization during early quantitative resuscitations^13^. Thus, hepatic dysfunction decreased lactate elimination. In cases of insufficient lactate elimination, lactate generation has a large effect on blood lactate level, potentially explaining the significance of lactate clearance in hepatic dysfunction.

In this study, delta base excess, which reflects the bicarbonate level at the end of lactate metabolism, was not associated with 28-day mortality in both the TBIL ≥ 2 mg/dL and TBIL < 2 mg/dL groups. High base excess reflects increased levels of non-volatile acids, including lactate, in sepsis^29^, whereas low base excess predicts increased lactate levels^30,31^. However, base excess is affected by other abnormalities such as metabolic acidosis (ketoacidosis, renal tubular acidosis, and uremia), acute respiratory acidosis, and changes in hemoglobin levels, all of which can occur in critically ill patients^32^. The application of the change in base excess as a prognostic marker for septic shock may be affected by these complex acidosis mechanisms.

Conversely, delta anion gap was significantly associated with altered mortality and lactate clearance. The anion gap, which simply reveals ion balance, reflects the level of nonvolatile acid more directly compared to the base excess, calculated using pH and bicarbonate level^33,34^. This explains the significant correlation between lactate clearance and delta anion gap, which in turn indicates the significant association between delta anion gap and altered 28-day mortality because lactate clearance is significant associated with 28-day mortality.

In the analysis of the derivation cohort, increased lactate clearance was also associated with decreased 28-day mortality in both Cre ≥ 2 mg/d group and APACHE II score ≥ 35 group. In the validation cohort, the association was just about significant in APACHE II score ≥ 35 group. Furthermore, the point estimate for 28-day mortality in lactate clearance in APACHE II score ≥ 35 group (0.86) was lower than in the TBIL ≥ 2 mg/dL group (0.89). It may indicate the lactate clearance being beneficial also in patients with severe disease, while no previous study showed benefits of lactate clearance as a prognostic value for mortality in patients with higher severity of sepsis or septic shock. Further studies of larger scale will be required to focus on the association between severities and lactate clearance in septic shock patients.

Our study has few limitations. First, it was a retrospective study; however, hepatic dysfunction had the same effect on both septic shock cohorts. Second, we defined hepatic dysfunction as TBIL ≥ 2 mg/dL based on the Brussels criteria; however, there are different published criteria or cutoff values for defining hepatic dysfunction. Finally, lactate clearance is associated with survival in not only patients with increased bilirubin levels but also in those with high APACHE II score and increased creatinine levels in the derivation cohort, which shows that increased lactate clearance is beneficial in all patients with increased severity and evidence of organ dysfunction.

## Conclusions

In septic shock patients with hepatic dysfunction (TBIL ≥ 2 mg/dL) or high APACHE II score (≥ 35), increased lactate clearance was correlated with decreased 28-day mortality. The findings of this study highlight the importance of hepatic dysfunction and severity in lactate clearance when considering lactate clearance as a clinical parameter.

## Supplementary Information

Supplementary Figure 1.

Supplementary Figure 2.

Supplementary Figure 3.

Supplementary Information.

## Data Availability

The datasets used and analyzed during the current study are available from the corresponding author upon reasonable request.
