# Supplementary figures and images for: Significance of lactate clearance in septic shock patients with high bilirubin levels
Source: Sci Rep. 2021 Mar 18;11:6313. doi: 10.1038/s41598-021-85700-w (PMC7973422; doi:10.1038/s41598-021-85700-w)

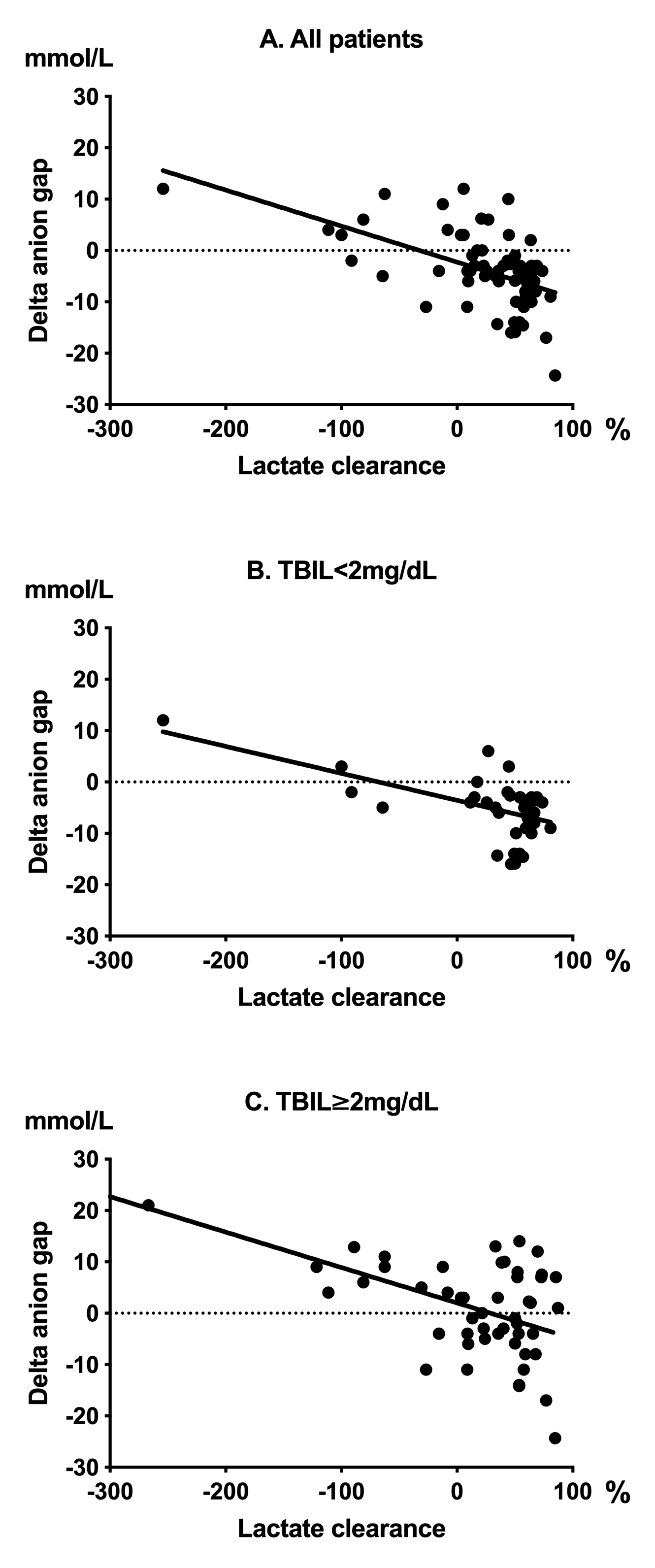

Supplement: Supplementary file 1 — Supplementary Figure 1. [file 41598_2021_85700_MOESM1_ESM.tiff]

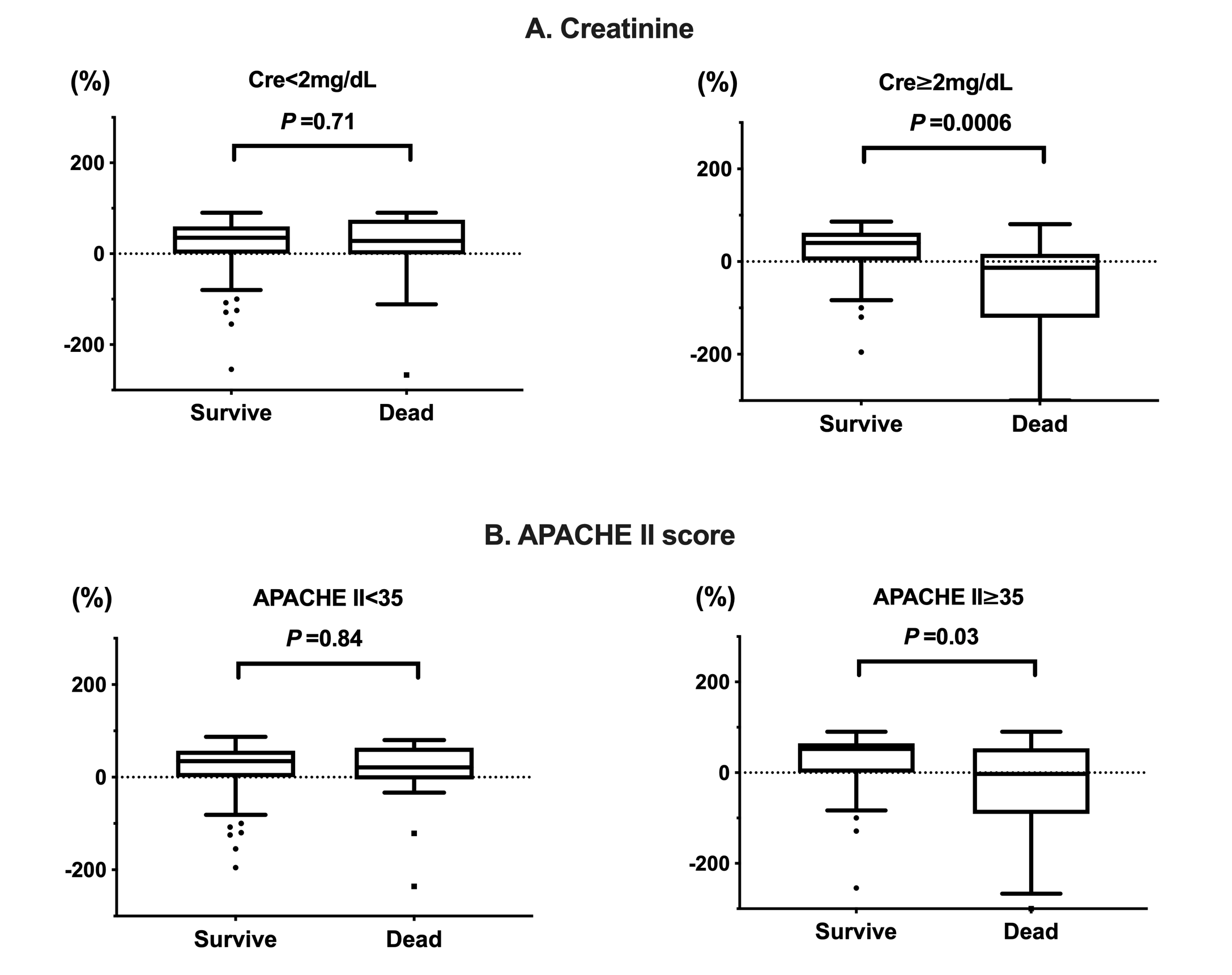

Supplement: Supplementary file 2 — Supplementary Figure 2. [file 41598_2021_85700_MOESM2_ESM.tiff]

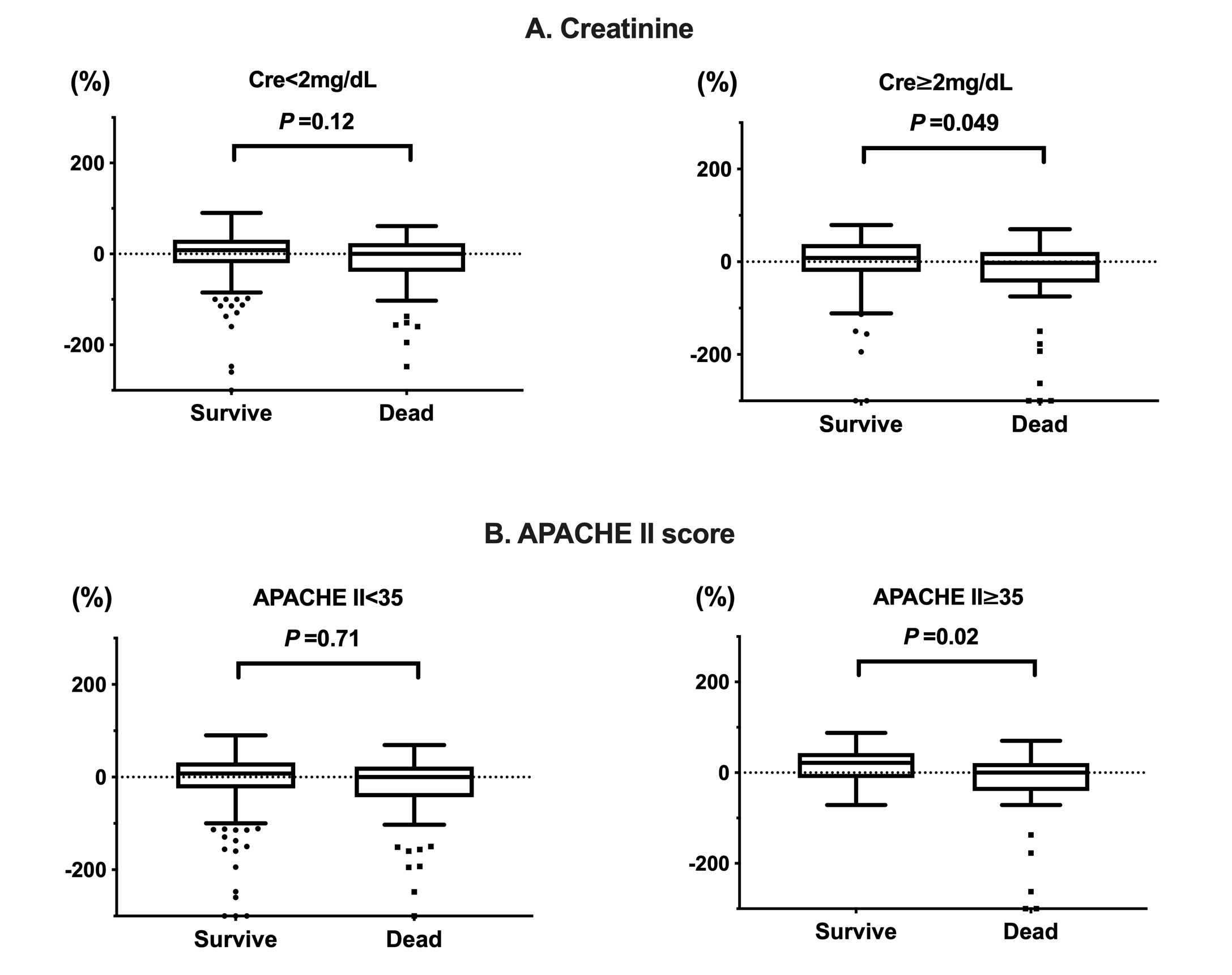

Supplement: Supplementary file 3 — Supplementary Figure 3. [file 41598_2021_85700_MOESM3_ESM.tiff]
