# Supplementary material for: Significance of lactate clearance in septic shock patients with high bilirubin levels
Source: Sci Rep. 2021 Mar 18;11:6313. doi: 10.1038/s41598-021-85700-w (PMC7973422; doi:10.1038/s41598-021-85700-w)
Supplement: Supplementary file 4 — Supplementary Information. [file 41598_2021_85700_MOESM4_ESM.pdf]

Supplemental Digital Content

Significance of lactate clearance in septic shock patients with high bilirubin levels.

Authors: Nozomi Takahashi MD PhD [1], Taka-aki Nakada MD PhD [1], Keith R. Walley MD [2], James A. Russell MD [2]

eTable 1. Multivariate logistic regression analysis for 28 days mortality in different TBIL levels (CHIBA cohort)

	Lactate clearance -per 10%	
	Odd ratio (95%CI)	P value
TBIL≥1mg/dL	0.93 (0.88-0.99)	0.016
TBIL≥2mg/dL	0.88 (0.80-0.97)	0.0075
TBIL≥3mg/dL	0.89 (0.81-0.99)	0.026
TBIL≥4mg/dL	0.97 (0.81-1.16)	0.76

Multivariate analysis was adjusted by age, sex, and APACHE II score

eTable 2. Multivariate logistic regression analysis for 28 days mortality stratified by Creatinine levels, platelet count and APACHE II score (CHIBA cohort)

	Lactate clearance -per 10%	
	Odd ratio (95%CI)	P value
Creatinine<2mg/dL	1.01 (0.94-1.08)	0.76
Creatinine≥2mg/dL	0.88 (0.81-0.95)	0.00069
Platelet<80 *10^3/mm^3	0.95 (0.90-1.01)	0.096
Platelet≥80 *10^3/mm^3	0.95 (0.88-1.03)	0.22
APACHE II score<35	0.96 (0.89-1.04)	0.37
APACHE II score≥35	0.93 (0.87-0.98)	0.013

Multivariate analysis was adjusted by age, sex, and APACHE II score

eTable 3. Multivariate logistic regression analysis for 28 days mortality stratified by Creatinine levels and APACHE II score (VASST cohort)

	Lactate clearance -per 10%	
	Odd ratio (95%CI)	P value
Creatinine≥2mg/dL	0.99 (0.97-1.02)	0.66
APACHE II score≥35	0.86 (0.74-1.00)	0.051

Multivariate analysis was adjusted by age, sex, and APACHE II score

Figure legend

eFigure 1. The correlation between lactate clearance and delta anion gap

Panel A. All patients, Panel B. TBIL<2mg/dL, Panel C. TBIL≥2mg/dL

eFigure 2. Lactate clearance between survivors and non-survivors in the derivation cohort

Panel A. Creatinine, Panel B. APACHE II score

eFigure 3. Lactate clearance between survivors and non-survivors in the validation cohort

Panel A. Creatinine, Panel B. APACHE II score
